# The Digital Phenotyping Project: A Psychoanalytical and Network Theory Perspective

**DOI:** 10.3389/fpsyg.2020.01218

**Published:** 2020-07-15

**Authors:** Rémy Potier

**Affiliations:** Department of Psychoanalytic Studies, Institute of Humanities, Sciences and Societies, University of Paris, Paris, France

**Keywords:** digital phenotyping, smartphone, psychoanalysis, data, case formulation, network theory

## Abstract

A new method of observation is currently emerging in psychiatry, based on data collection and behavioral profiling of smartphone users. Numerical phenotyping is a paradigmatic example. This behavioral investigation method uses computerized measurement tools in order to collect characteristics of different psychiatric disorders. First, it is necessary to contextualize the emergence of these new methods and to question their promises and expectations. The international mental health research framework invites us to reflect on methodological issues and to draw conclusions from certain impasses related to the clinical complexity of this field. From this contextualization, the investigation method relating to digital phenotyping can be questioned in order to identify some of its potentials. These new methods are also an opportunity to test psychoanalysis. It is then necessary to identify the elements of fruitful analysis that clinical experience and research in psychoanalysis have been able to deploy regarding the challenges of digital technology. An analysis of this theme’s literature shows that psychoanalysis facilitates a reflection on the psychological effects related to digital methods. It also shows how it can profit from the research potential offered by new technical tools, considering the progress that has been made over the past 50 years. This cross-fertilization of the potentials and limitations of digital methods in mental health intervention in the context of theoretical issues at the international level invites us to take a resolutely non-reductionist position. In the field of research, psychoanalysis offers a specific perspective that can well be articulated to an epistemology of networks. Rather than aiming at a numerical phenotyping of patients according to the geneticists’ model, the case formulation method appears to be a serious prerequisite to give a limited and specific place to the integration of smartphones in clinical investigation.

## Introduction

A new method of observation is currently emerging in the psychiatric field, based on data collection and behavioral profiling of smartphone users. Numerical phenotyping is a paradigmatic example. This behavioral investigation method refers to the use of computerized measurement tools in order to collect characteristics of different psychiatric disorders. This method seems all the more feasible and promising as the number of smartphone users is constantly growing worldwide.

In a context, where there are countless concerns about privacy and data ownership, such method raises major ethical issues. These concerns immediately engage researchers to thoroughly reflect on all issues underlying clinical investigation *via* digital technology, without undermining past mistakes.

Thence, it is necessary to contextualize the emergence of these new methods and question their promises and expectations. From such contextualization, the investigation method relating to digital phenotyping can be questioned and its potential benefits and dangers better identified. New observation methods and orientations can also constitute an opportunity to test psychoanalysis in a fruitful way. What is the reality of this data and how can we not reduce the meaning of the traced behaviors thus collected? What methodological lessons can be drawn from mental health issues? What use should be made of a tool and with which purpose?

The use of new techniques for clinical observation can be articulated to current mental health controversies. It is in this context that digital phenotyping as a technique and research program can be fully understood.

In addition, it will be necessary to highlight what contributions psychoanalysis can bring to the understanding of the issues related to digitization, as well as the new possibilities offered by these techniques. The position of psychoanalytical research in this type of program can thus be established.

## New Orientations in Psychopathology in a Digital Context

### Controversies and Mental Health: Context for a New Method of Investigating Mental Illness/Disorders

Scientific results of research on psychological disorders and psychopathological problems always appear at the margin of the development of general medicine according to the ideal of Evidence Based Medicine. The register of evidence, despite decades of investigation, does not reach the level expected in order to be seriously compared with other fundamental research areas such as neuroscience or genetics for example. Ethical questions remain, and an analysis of speeches and content allows at a minimum to relativize or intentionally encourage neuroskepticism in the field of mental health. For the philosopher Forest, the “question” to be asked is “that of the trust we can rationally place in today’s brain sciences when, in turn, they speak to us about ourselves” (Forest, 2015a, p. 10). Neuroskepticism consists in questioning the validity, usefulness, or harmlessness of neuroscientific knowledge according to its contexts of application and does not lead to an anti-scientific stance. To make science is not only to create methods or theories, but also to set up “collective rules” according to Merton (1973). Organized skepticism is understood according to this objective; which means that skepticism is not science’s enemy but its condition. This philosophical position is therefore to be used as an instrument in the service of research in psychopathology.

Indeed, recent publications encourage us to step back and recognize the rhetorical effects of discourses bordering on neuro-realism (Racine et al., 2010). Some bold and scientifically promising hypotheses often emerge in an explosive manner in the international context. However, while there is no doubt that the development of genetics makes it possible to rely on discoveries that lead to more targeted clinical work (Potier and Putois, 2018), it is not certain that psychological research can claim its scientific significance, if only in the design of quantitative studies (Ioannidis, 2019). The phenotyping of mental illness is clearly not a given, and the project is still controversial. For example, the discovery of the 5-HTTLPR gene involved in depression may raise some instructive concerns (Risch et al., 2009; Clarke et al., 2010). These meta-analyses have already highlighted some doubts or limitations. The narrative supported in some of these studies is overly optimistic about the results. Over the past 2 decades, articles have attempted to show the presence of 5-HTTLPR in other neuropsychiatric conditions (e.g., stroke; Mak et al., 2013). This is shown by an important article by Border et al. (2019), which provides an analysis with a decisive methodological scope. According to the Border et al. neither 5-HTTLPR nor any of the 17 other comparable “depression genes” had an effect on depression. These genetic researchers have developed an infrastructure to analyze samples of hundreds of thousands of people using standardized techniques. According to them, identifying depression genes would require samples of about 34,000 people to be reliably detected, so any study involving less than 34,000 cases and specific genes is certainly a false positive. They argue that isolated genes are generally less important than we think.

The reductionist temptation from mental disorders to strictly neurological pathologies also seems to be overwhelming. However, the media coverage of the promising discoveries has brought false certainties to the general public (Racine et al., 2005, 2006, 2010). A neuro-ethical perspective on strictly neuroscientific uses and paradigms in mental illness offers a cautious look at the promises of contemporary psychiatry (Gonon, 2009; Gonon and Konsman, 2011; Gonon et al., 2012). Controversies remain despite what some studies may suggest, which are too affirmative, so that a strictly and exclusively biological approach to psychiatry may appear as a speculative bubble (Gonon, 2011). The question of referring to reliable information arises in a major way, as in the example of attention deficit hyperactivity disorder (ADHD) (Ponnou et al., 2019). What if mental disorders are not just brain disorders? This classic question needs to be asked again to open up the field of contemporary research in the context of mental health honestly and faithfully. A recent study by Borsboom et al. (2019) makes it possible to move forward on this issue and make promising proposals after having been able to demonstrate the failure of neurological reductionism in psychiatry to account for clinical specificities in context (Ioannidis, 2019). A neuro-skeptical approach would provide an epistemological framework for the complex and multifactorial issues facing psychopathological research. Indeed, far from excluding the contribution of neuroscience, such a perspective makes it possible to avoid blindness and to treat problems according to their concrete cause. Such a limitation applies *a fortiori* to psychoanalysis, which, when it surpasses its field can venture into theoretical approximations and projections. Finally, epistemological questioning must preside over the innovations and boldness necessary for research in order to make the most of what each method can shed light on in its own right.

### The International Research Framework

Since the release of the Diagnostic and Statistical Manual of Mental Disorders, Fifth Edition (DSM-5), the international controversy concerning orientation has raised suspicion about any desire for innovation (Demazeux, 2013; Sedler, 2016). Relying on data from the scientific literature is definitely not a guarantee of neutrality and objectivity. The funding effect is a problem that should not be minimized if we are concerned with the ethical issues of both research and clinical practice. In addition, this version of the manual presents problems regarding its standard’s design and contains biases that do not allow its use within certain contexts and cultures. Indeed, expanding definitions of illness imply a reconfiguration of the underlying category of normality (Sweet and Decoteau, 2018). Similarly, the cultural and contextual specificities of the clinical field remain poorly thought-out (Vanheule, 2017; Bredström, 2019). Many clinicians are abandoning the DMS as a clinical benchmark because they are confused by the myriad of specifications that result from it.

In parallel with the DSM other influential initiatives are becoming important in the field of mental health. The DSM and International Classification of Diseases (ICD) have traditionally conceptualized psychological disorders categorically, but in recent years limitations of polythetic diagnoses based on combinations of symptoms have become increasingly apparent (Bornstein, 2019). Furthermore, dimensional models of psychopathology have emerged in an influential way. Typically, these models conceptualize psychological syndromes using a series of trait dimensions, with patients being assigned severity ratings that are combined into a trait profile to capture the central elements of a particular disorder. The Hierarchical Taxonomy of Psychopathology (HiTOP) is one of the dimensional models most representative of these initiatives (Kotov et al., 2017, 2018; Krueger et al., 2018; Conway et al., 2019).

The Research Domain Criteria (RDoC) project, launched in 2009 in the United States by the National Institute of Mental Health (NIMH), is emerging as a new research direction. The RDoC project, dedicated to research, contrasts with the DSM by focusing on the dimensions of normal brain functioning, at the intersection of genetic research, cognitive neuroscience, and behavioral sciences.

The RDoC seems to be convinced that mental illnesses are brain disorders. The RDoC project proposes to focus on psychological constructions that show a good affinity with neurobiology. This change of rules and the RDoCs thus converge toward a goal, which is to orient clinical research with the contributions of biological and cognitive sciences (Demazeux and Pidoux, 2015). The aim of this project is to develop a classification of psychopathology or “biopsychopathology” (Kozak and Cuthbert, 2016, p. 288) Non-biomedical dimensions of human suffering are largely left out of consideration. The RDoC project does not take into account the fact that people are signifier-using creatures who have a reflexive relation to their own mental life and the world they live in, and who attempt to make sense of reality (Vanheule, 2017). The RDoC project seems to announce an approach to diagnosis in which a person’s self-reflexive relation to his own suffering is left out. Indeed, there seems to be a rupture with any form of subjectivism behind this project (Kozak and Cuthbert, 2016; Hsin et al., 2018). For Frances, however, human understanding of psychopathology and its treatment methods has not really improved (Frances, 2014). Indeed, according to him, the NIMH has evolved into a “brain institute” rather than a “mental health institute.” Also, at this stage, the RDoC project would have lost its mind, Frances described it as “mindless.” Betting on brain research is certainly necessary for a better future, but it should not dominate current needs so completely.

For Vanheule, the RDoC project should not be definitively entirely opposed to the exploration of subjectivity (Vanheule, 2017). The five respective domains and sub-domains of the RDoC matrix could also be studied from a contextual and thematic point of view. Only, for this to be effective, theoretical and methodological paradigms that are no longer strictly quantitative and experimental should be employed. However, following the criticisms of the DSM and the observation of certain limitations linked to scientific investigations in recent decades, new methods and programs are being developed to support the improvement of digital technology, a process which deserves to be examined, taking into account the recent extrapolations identified in our field.

### Digital Phenotyping Programs

The RDoC criteria framework is intended to provide a useful roadmap for organizing, guiding, and directing new data and moving toward the digital phenotyping project (Torous et al., 2017). This ambition is fully in line with the overall project.

Recent discoveries in the fields of machine learning (ML), computer adaptive testing (CAT), momentary ecological assessment (EMA), or experience sampling method (ESM) have opened the way to new modalities of investigation in patient observation. In the field of psychology, these clinical investigation modalities have been met with great interest, since the first experiments (Hormuth, 1986; Csikszentmihalyi and Larson, 1987; Stone and Shiffman, 1994) relied on the development of smartphones (Trull and Ebner-Priemer, 2009). However, according to researchers who have been developing these methods for more than 20 years, they improve our understanding of how psychopathological symptoms manifest themselves over time in daily life using embedded technologies.

With smartphones, these methods open up new and unexpected tracking dimensions for researchers who could offer technical support for real progress in psychiatry. Mobile and connected devices such as smartphones and portable sensors can facilitate the collection of new data for psychiatry, both to caregivers (Zhang et al., 2016a,b) and the population being cared for. Can certain aspects of the use we make of technology interfaces serve as data for potential diagnoses? (Insel, 2017, 2018). Can a person’s clinical data be linked and analyzed with online activity and behavior data to create a unified and nuanced view of mental disorders? This is what some current researches seem to believe and defend, defining the digital phenotype and describing in more detail the opportunities and challenges of integrating this data into health care (Jain et al., 2015). Early studies using these methods hold the promise of providing a more complete and nuanced view of disease experience. But defined in this way, the disease experience is limited to the behavioral traces materialized by the numerical data, much more so than the disease experience (Canguilhem).

The role of numerical phenotypes in diagnosis would go beyond surveillance and early detection. Numerical phenotypes redefine disease expression based on the behavioral experiences of individuals that can be digitized, thereby increasing the ability to classify and understand the disease (Insel, 2017, 2018).

Digital phenotyping by smartphone could potentially provide the psychiatry field with a wealth of data on disease phenotypes that could offer new leverage in each of this field’s areas (Onnela and Rauch, 2016). Although the scope and scale of the data allow for new approaches of health, data alone are not sufficient.

Over the past 5 years, we would at one point be at the crossroads of psychiatry, technology, and quantitative science through embedded technologies. Digital phenotyping methods are emerging in this context. This innovative and evolving aspect is highlighted by some researchers, who see in it the promise of a significant contribution to the field of psychiatry (Insel, 2017, 2018).

Digital phenotyping expectations suggest that passively collected behavioral data from smartphones provide an evolving and currently underutilized opportunity to monitor patients for possible warning signs of relapse (Barnett et al., 2018; Huckvale et al., 2019).

The use of digital phenotyping has been particularly developed in the field of addiction (Skinner et al., 2017; Ferreri et al., 2018) and would also be useful to guide clinical diagnosis and screening for autism spectrum disorders (ASDs) based on tweets analysis (Hswen et al., 2019). ASD is a developmental disorder that affects communication and behavior. People with ASD have difficulty with communication and interaction with other people, restricted interests and repetitive behaviors, and symptoms that hurt the person’s ability to function in school, work, and other areas of life. In fact, their study aims to explore the feasibility of using the web-based social media platform Twitter to detect psychological and behavioral characteristics of self-identified persons with ASD. As in the field of trauma, an exploration of post-traumatic stress disorder (PTSD) conditions through smartphones is promising (Bourla et al., 2018c). The field of psychosis has also been investigated, in particular the management of schizophrenia (Hswen et al., 2018; Torous and Keshavan, 2018; Wisniewski et al., 2019). In the field of perinatal psychiatry, smartphone applications could be a novel method to addressing the gap in the provision of postpartum care services, providing psychoeducation, and improving maternal parental self-efficacy for childbearing women (Fealy et al., 2019).

The development of such smartphone applications requires significant private investment. The modeling of relevant data requires, in addition to serious guidance in the world of raw data, the talent of innovative data scientists in the processing and modeling of these data. Focused on the traces of digital phenotyping involves collecting sensor, keyboard, and voice and speech data from smartphones to measure behavior, cognition, and mood (Insel, 2017). From this perspective, the disorders in their diversity can be investigated, in particular mood disorders (Bourla et al., 2018a) or sleep disorders (Teo et al., 2019). The primary research application of embedded technologies to investigate these disorders appears to be depression (Rajagopalan et al., 2017). The process involves exploring the detection of everyday life behavior based on sensor information to identify subjects with clinically significant levels of depression. The challenge is also to explore the potential of a context-sensitive intervention to provide *in situ* support to people with depressive symptoms (Wahle et al., 2016). In this field, interaction with the telephone keyboard would be significant for both depression and mania digital phenonotyping (Zulueta et al., 2018). Numerical phenotypes are then a strategy to predict relevant outcomes of mood disorders, including relapse, recurrence, cognitive decline, and functional impairment (Brietzke et al., 2019; Jacobson et al., 2019). But despite technological advances and the introduction of many mobile phone apps that claim to relieve depression, there are still significant gaps in knowledge about what applications actually measure, and what and how they correlate with psychometric questionnaires (Moukaddam et al., 2019). In fact, the digital phenotyping concept is rapidly expanding and becoming increasingly used in child and adolescent psychiatry (Sequeira et al., 2019). Smartphones and similar devices can not only lead to the development of interventions (Doryab et al., 2019) but also contribute to suicide prevention (Kleiman et al., 2018).

This work aims to contribute to the advancement of the complex issues addressed in mental health by recognizing the need for a holistic approach within the framework of general psychopathology, taking advantage of interdisciplinary work on data, as well considering the different parameters involved in the disease. A more holistic approach – encompassing both biological and non-biological approaches – is likely to produce greater contributions to the understanding of the nature and basis of psychopathology and to accelerate the development of improved interventions for patient suffering.

Digital phenotyping will involve the collection of massive amounts of individual data and the potential creation of new categories of health data and participation in risk assessment. Since existing ethical and regulatory frameworks for the provision of mental health care do not clearly apply to digital phenotyping, the need to consider all possible ethical, legal, and social implications is already recognized as essential (Martinez-Martin et al., 2018). According to Martinez-Martin and colleagues, transparency, informed consent, privacy, and accountability are the four main ethical guarantees that must guide development. It is indeed important to consider these issues at the early developments of this new approach, so that its promises are not limited by adverse effects or unintended consequences. Nevertheless, in order to feed the ethical debate and not limit this new method of observation to reductionism or neo-naturalism, it is necessary to open up to interdisciplinary exchanges about data and to take into consideration the limitations of these data to define subjectivity.

To recognize in what ways the consideration of the subjective dimension questions these data appear quite essential. In this respect, it is already significant that the first investigations using this method have given rise to the concept of digital phenotyping. The exclusive temptation of the biological model and the genetic ideal appears in this concept, perhaps as a potential limitation of data analysis.

In order to discuss the heuristics of this method and to cross-reference observations, the contrast that appears between these innovations and the psychoanalytical analysis of the clinical field and the methods associated with digital tools can nourish a fertile tension. What and how can we learn from the psychoanalytical clinic about a patient’s relationship to their own data? How can the psychoanalytical approach question and contribute to this new field of research investigation?

## The Contribution of Psychoanalysis to the Understanding of Digital Technology

To better understand what digital phenotyping involves and how it can be applied to improve care, sophisticated methods for rigorously capturing and analyzing the various digital health data flows will be required. However, data from analytical experience are essential to understanding the dimension of transfer in the therapeutic relationship, precisely at a time when technology occupies a place in this relationship. Raising the novelty of what characterizes digital technology also invites the clinician to protect themself from wild projections resulting from their own uses or even their ideals as a researcher. Nevertheless, the mobile phone is an anthropologically unavoidable object and probably unexpected in terms of its research potential (Ferraris, 2014).

### Daily Virtual Clinics

The digital phenomenon, as a contemporary reality, challenges psychoanalysts in their practice. This phenomenon can be identified in research at the international level, as evidenced by psychoanalytical literature involving network links in their reflection and analysis. These new digital forms of sociability (Casilli, 2010) interest psychoanalysts from the different, new prisms that practice and contexts offered to analysis, in the singularity of each case. Whether it is the question of digital culture itself, that of unease in culture, or that of the new practices that are induced by the new modalities of care requests, epistemological issues are also one of the central points that trigger reflection in this field.

Many psychoanalysts propose contributions, based on international collaborations, to study the virtual’s impact on our society and the uses psychoanalysts can make of it (Laszig and Eichenberg, 2003; Migone, 2013; Scharff, 2013; Eichenberg and Hübner, 2018; Thorwart, 2019). Further research on the online psychotherapeutic framework leads to heuristic reflections and new projects. For example, the book *Psychoanalysis Online*, edited by Scharff, is based on an international collaboration of psychotherapists and psychoanalysts who study the impact of the virtual on our society and the uses of communication that psychoanalysts can make of it. Concerning social media itself, German authors have recently been relaying the issue. Eichenberg and Hübner, researchers and psychoanalysts in Berlin, propose examination of how the development of modern media affects psychoanalytical theory and practice as much as social relations and other areas of mental health. In particular, psychoanalytic treatment on the Internet creates an ambivalence between doubts and criticisms of the use of new media in psychoanalysis. Instead of a one-sided discussion of the possibilities and limits of online psychoanalysis, the authors argue for an empirical explanation of the psychoanalytical process of communication on the Internet. They propose useful implications and practical recommendations for psychoanalytic treatment and focus on statutory rules and ethical aspects as well as indications and contraindications for online sessions in the conduct of therapeutic procedures. We are currently launching an international study on online care during the pandemic, which may soon provide further insights into these issues.

The question of transference, particularly through online interviews, is being addressed. On the occasion of these new social practices, certain key concepts of psychoanalysis can be studied in a renewed way. The concept of compulsion of repetition in particular can appear in a virtual act that is significant for the patient. The immediate properties of digital technology are also important to take into account as it invites magical thinking and symptomatic manifestations that can be identified by patients themselves. This expertise in psychoanalysis reflects the particular link that each subject has with digital technology. This aspect is absent from studies on numerical phenotyping and should be included as a programmatic aspect in order to avoid unexpected problems (Martinez-Martin et al., 2018).

Before expanding this clinical exploration research *via* smartphones, it is important to refer to critical and sensitive data reported by psychoanalysis in particular. For example, Turkle’s work (Turkle, 2016, 2017) first assesses in great detail how new technologies have opened up complex networks of connections. In reclaiming conversation, she insists that forms of digital communication are generally not additive, but reductive. Texts, emails, and even video games have emerged from the aspiration to be fully embodied forms of communication, but almost always with something less. As a clinician, we are therefore forced to reflect on a number of fundamental questions about the changing nature of our sociality and the new modalities of induced care. Excess data could also result in an abandonment of contact with the patient, which is a significant risk for the financial savings expected from these technologies. Already, bots meet the most standard patient demands from apps available on mobile platforms. Turkle notes in her interviews and in her clinical practice that people invest themselves anxiously through digital exchanges in relationships and not in a serene and constructive way. She uses the term “edited life” to describe this phenomenon.

The use of “texting” (sms or instant messaging), is thought by Turkle as a means of communication without implication, allowing to communicate without engagement, without taking risks, without needing to reveal oneself, and without needing the authenticity of the other. This form of communication itself is sufficient. And yet, it produces a real dependence on the time flow of received messages, an irrepressible need to sneak a peek at the screen of your phone just in case. There is a compulsive need to “share,” electronically and instantly, to fully experience events and emotions, as if something was lost because you are “off-grid.” Turkle alerts us to this superficiality of the self, which needs a communicative reinforcement to give the thickness of reality to what one lives. However, behavioral data from keyboard interactions, numerical exchanges, etc., do not seem, in digital phenotyping studies, to take into account the alienation dimension. Therefore, care should be taken not to yield to these contemporary modalities of communication to the detriment of the actual clinical encounter. Once this dimension has been identified, the scope of these new emerging methods still needs to be defined, particularly within the overall framework of care. At the same time, digital sociality holds unsuspected surprises that should not be overlooked under the guise of a misonestic principle. Indeed, the Covid-19 pandemic has had unexpected implications, specifically the digitalization of relationships with the other, which brings its share of new questions and interests. New observations will no doubt allow us to reflect anew on the implications of digital technology.

### Digital Relational Modalities: Clinical and Ethical Considerations

Among the various ethical issues that must be taken into account, those raised by digital labor testify to sensitive clinical problems (Favero and Candellieri, 2017; Johanssen, 2018). The Freudian affect model can help us better theorize the emotional work involved in social media (Johanssen, 2018). Clinical data provide a relevant subjective view of what is emotionally involved in digital interactions, which should be considered as one of the dimensions of any post-analysis of the data produced by and with the support of the patient. Whether passive or not, these data are never neutral and can characterize an effective digital labor.

It is true that many psychoanalytical texts are critical of our contemporary civilization and apply the Freudian concept of “Discontent in Culture” in order to decipher its effects. According to this thesis, the current era is perverse because of a social paradigm facilitated by an explosive technological progression that would rapidly modify the erotic and social dimensions of human relations, particularly through smartphone apps, especially dating apps (Knafo, 2015). Nevertheless, this world view is subject to debate. Digital tools also offer individuals the opportunity to immerse themselves in a wider range of sexual, racial, or gender identities in a therapeutic way for themselves. These are all opportunities for the individual to return to a safer and more fun arena than the one that has been experienced before in their own history (Watts, 2017).

Thus, the intense psychic effects induced by the compulsive use of digital technology is a subject particularly explored by psychoanalysts who can provide precise data on the unconscious side and the singular meaning of digital traces (Tyminski, 2015; Vlachopoulou, 2018).

Some conceptual contributions from psychoanalysis could indeed prove to be particularly heuristic to help us think about the specific relational modalities of the digital realm. The concept of magical thinking (Tyminski, 2015) or the concept of “narcissism of minor differences,” (Potier, 2012) for example, testify of how the symptom has found its way into the paths offered by the possibilities of digital reality.

In addition, digital technology can be used in therapeutic mediation. Digital workshops and various digital media are now an integrative part of the psychologist’s tools in health care institutions (Haza, 2019). Thanks to this type of practice, it is possible to reflect more deeply about the psychological issues related to digital technology, particularly through the immersion experiences promoted by screens.

### The Contribution of Psychoanalysis to the Analysis of Data in Context

In the clinical context we are relentlessly “obliged to adapt our technique to these new conditions, we will have to give our theoretical doctrines the simplest and most accessible form” (Freud, 1919) by knowing how to carry in each contextually determined period, the cutting edge of Freudian discovery. Thus, investing in digital technology and its possibilities could also be an opportunity to observe, in an ostensibly visible way, how psychoanalysis can provide, from its practice and method of investigation, fertile support for interdisciplinary research, particularly in the field of mental health.

However, psychoanalysis always has to reinvent and position itself according to the theoretical and civilizational challenges of each era. This is how we can interpret one of Lacan’s famous quotations:

“Let whoever cannot meet at its horizon the subjectivity of his time give up then. For how could he who knows nothing of the dialectic that engages him in a symbolic movement with so many lives possibly make his being the axis of those lives? Let him be well acquainted with the whorl into which his era draws him in the ongoing enterprise of Babel, and let him be aware of his function as an interpreter in the strife of languages” (Lacan, 1953, p. 264).

Lacan was also able to let himself be challenged by cybernetics and the enigma of Turing’s machine (Saint-Jevin, 2017). In psychoanalytic research and in a completely different epistemological orientation, many psychoanalysts have examined the contribution of information theories and have proposed to use the computer to analyze analytical records for algorithmic processing (Dahl, 1974). Similarly, the computer’s computing power could be used for a textual analysis of the Schreber Case, a method that reveals unexpected and stimulating results (D’Dell and Weidman, 1993).

Models derived from information system theories make it possible to investigate the conditions of occurrence of psychological phenomena and not the intrinsic nature of the phenomena themselves. Nevertheless, in the 1980s, according to some authors, these information and systems models promised psychoanalytical theory contributions in phase with the contemporary scientific thought where it could have been possible to re-found metapsychology on this new theoretical basis (Peterfreund, 1980; Rosenblatt and Thickstun, 1984). These pioneering projects deserve to be thoroughly reworked at a time when technical devices are closer to patients.

The use of recording analysis is one of the advantages offered by smartphones today on which the digital phenotyping project is based. However, the potentiality of working on session recordings has already mobilized analysts in particular. Recording audio sessions and reviewing sessions can be a particularly useful technical parameter in situations of processing impasse (Robbins, 1988). Indeed, traditional technical elements as well as some of the constructive elements of the analytical process itself can be investigated in the aftermath, from linguistic analyses of verbatim (Dahl et al., 1978; Karp et al., 1993).

This use of computer technology for the detailed analysis of the therapeutic process at work in analytical sessions has been the subject of numerous developments since the mid-1970s and continues to inspire teams.

Indeed, the Ulm Psychoanalytic Process Research Study Group was able to highlight the experimental potential of psychoanalysis by focusing a number of structured research methods on a single case study (Thomä and Kächele, 1975). At the basis of the Ulm process conception lie empirical approaches allowing to identify when focal phases take place (Kächele, 1988). The work of this group has identified replicable methods, tools, and experiments that are quite inspiring for the analysis of relational interaction (Kächele et al., 2011).

Indeed, the Ulm Psychoanalytic Process Research Study Group highlighted the experimental potential of psychoanalysis by focusing a number of structured research methods on a single case study (Thomä and Kächele, 1975). At the basis of the Ulm process conception lie empirical approaches to identify when focal phases take place (Kächele, 1988). The work of this group has identified replicable methods, tools, and experiments that are quite inspiring for the analysis of relational interaction (Kächele et al., 2011). In the same perspective, Fertuck and colleagues developed the Reflective Functioning scale (RF), which is a narrative-based assessment of the capacity to coherently conceptualize one’s own and others’ subjective motivations, emotions, beliefs, and desires. They developed a computerized text analysis version (CRF) of the Reflective Functioning assessment system. A sample of clinical and non-clinical Adult Attachment Interviews (AAI) were utilized by the authors to develop their measure (Fertuck et al., 2004, 2012).

In this vein, Mergenthaler has developed the “Therapeutic Cycles Model” (Mergenthaler and Stigler, 1997; Fontao and Mergenthaler, 2002, 2008; Lepper and Mergenthaler, 2007, 2008; Benelli et al., 2012). Bucci and colleagues have done dictionary-based work that measures “Referential activity.” The Therapeutic Cycles Model (TCM) and Heidelberg Structural Change Scale (HSCS) (OPD Task Force, 2008) were also used to investigate therapist-patient dynamic processes across sessions of psychotherapy (McCarthy et al., 2011). In the same perspective, More specifically, the Weighted Referential Activity Dictionary (WRAD) is a dictionary (word list) containing 696 items, with weights ranging between −1 and +1, used for computer modeling of a psycholinguistic variable, referential activity (RA), in spoken and written languages (Bucci, 2000, 2013, 2018; Bucci and Maskit, 2004).

In order to develop a more universal model of the referential activity, recent research has been carried out. According to this research, the process of verbal communication of emotion as this occurs through the phases of the referential process, including arousal of an emotion schema; detailed and specific descriptions of images and episodes that are exemplars of emotion schemas; and reflection and reorganization, which may include emotion labels and other types of categorical terms (Bucci et al., 2016). All of which, since psychoanalysis, could enrich algorithmic modeling for the analysis of smartphone data. Technical possibilities are now being explored and show promising success stories. In the field of medicine, for example, text analysis is making inspiring advances. Mullenbach and colleagues propose an attentional convolutional network that predicts medical codes from clinical text. Their method aggregates information across the document using a convolutional neural network and uses an attention mechanism to select the most relevant segments for each of the thousands of possible codes. Through an interpretability evaluation by a physician, they show that the attention mechanism identifies meaningful explanations for each code assignment (Mullenbach et al., 2018).

This dimension could be a valuable contribution to reflect on the potentials and limitations of clinical observation *via* smartphones.

Other experiments could be very useful for detailed data mining, particularly to evaluate psychotherapeutic management and its role in overall treatment. Scharf and colleagues developed the analytic process scales (APS) (Waldron et al., 2004). The APS was a measure designed to assess the degree of “analytic process” occurring in psychotherapy. The analytic process scales (APS) was developed by a team of experienced psychoanalysts and may be of particular interest to researchers who wish to study psychotherapy process or psychoanalysis specifically. This tool measures in a manner that allows it to assess psychodynamic process across various forms of psychotherapy. Studies on the measurement of verbal interventions in a psychotherapeutic context are also a valuable source (Gumz et al., 2015), whose experiments and results should guide the research initiated today *via* embedded technologies.

Other studies, particularly on relational dyad, provide very specific elements concerning the interpretation of voice in relation to attachment issues (Beebe et al., 2000). Beebe and colleagues highlight the importance of time in the coordination of vocal rhythms and attachment processes within two-person dyads. Different patterns of vocal timing coordination predict different emotional climates. This could be very useful in conceptualizing the emotional climates in a psychotherapeutic relationship.

Thus, there are strong arguments in favor of a holistic approach in psychopathology that allows an interaction between the bio-psycho-social dimensions. In such a conception, psychoanalysis has its full place. For example, there is a way of thinking about the brain that is increasingly common in cognitive science, mainly as a prediction machine. The “predictive brain” approach represents a major paradigm shift from the brain-computer analogy of cognitivism in the 1970s. The brain does not passively wait for its “inputs” to “process symbolic representations” and produce “outputs.” Rather, it is constantly trying to make inferences from the physical disturbances to which its senses are subjected in an attempt to understand their causes. Friston’s work on free energy meets and inspires contemporary psychoanalysis in a heuristic way (^63,61^; Friston and Stephan, 2007; Friston and Kiebel, 2009). Friston and Carhart-Harris have indeed linked Freud’s theoretical propositions to data from neuropsychology, neuroimaging, and psychopharmacology (Carhart-Harris and Friston, 2010, 2019). This Bayesian conception has fundamental consequences to be taken into account for the analysis of data collected *via* smartphones insofar as they testify to a path through traces. The predictability of relapses, for example, could be based on such a design. The main concepts of free energy (FE) neuroscience run parallel to those of Freud’s Scientific Psychology Project (Holmes and Nolte, 2019). Mental disorders are thus caused by computational complexity as well as by mechanisms such as synaptic pruning (Hopkins, 2012, 2016). The main concepts of FE neuroscience developed by Friston and his colleagues parallel those of Freud’s Scientific Psychology Project. Mental disorders are thus caused by computational complexity as well as by mechanisms such as synaptic pruning. (Hopkins, 2012). Hopkins suggests that conflict can be potentially quantifiable as free energy from an EFP perspective (Hopkins, 2016). However, the implications that this formalization has for psychoanalysis have been thoroughly explored (Connolly and van Deventer, 2017; Connolly, 2018), which implies an interesting contribution to consider what could be collected as data from digital phenotyping methods. In this vein, it would be necessary to explore the contribution of this epistemology to psychoanalysis and the usefulness that an epistemology of systems theory – in particular hierarchical recursive description – can have in achieving this objective.

## Discussion

This article aims to reflect on the framework in which the emergence of new methods of investigating mental illness *via* smartphones is becoming a fully-fledged program and a serious orientation of contemporary psychiatry. The examination of the digital phenotyping projects appears as paradigmatic of this new orientation. These projects arise in a context, which needs to be highlighted in order to specify the investigation framework of this new method within the limits of a strict pragmatic and methodological realism. Thus, the particularities of the field related to mental health reveal that the subjective part of the subject’s relationship to their data must be taken into account. This is what a case formulation (Thurin, 2017; Vanheule, 2017; Juskewycz et al., 2018) can reveal, whose methodology could inspire good practices in the use of smartphones to complement the evaluation. Similarly, it could be fruitful to take advantage of previous analyses and the difficulties associated with reductionism in psychopathology.

### From Phenotype to Case Formulation

Digital phenotyping projects have the merit of revealing the potential of smartphones for the clinical investigation of mental disorders and proposing to lay the foundations for this research. As we use our smartphones and interact with the digital world on a daily basis, we leave behind digital fingerprints. These traces, which reflect our behavior, are what can be used for measurement. The technical properties of the smartphone are exceptionally promising for research in natural environments. However, the exploitation of these traces is still risky. Global Positioning System (GPS) location and mobility, battery charging frequency, voice and speech modes, SMS length or frequency, interaction with the keyboard, call log, frequency of visits to an application, ergonomic organization of our smartphone, interaction with the screen, navigation path on the Internet, and influence of algorithms on behavior, or even frequency of application updates – all this data can constitute digital phenotyping according to its given definition. However, there is a major difference between the potential for recording digital traces that characterize the mobile phone and the meaning given to the resulting data. On this point, moving from psychoanalysis toward these new methods of observation in an open mind would consist of taking into consideration this issue of data interpretation and therefore to engage in interdisciplinary research and to carry out research work around the data.

The term “phenotype” expresses the hope for a behavioral science based on personal data that can have the reliability of genomics. This would probably require establishing a huge cohort of patients, something that current projects are far from being able to build. Although, there are many portable systems and mobile applications available to characterize, analyze, and monitor mental health conditions, they have not been clinically evaluated on patients on a large scale for their accuracy and effectiveness (Liang et al., 2019). Finally, it seems the term digital phenotyping engages the expectations of some researchers more than the research potential resulting from the consideration of smartphone data for clinical observation. Researchers are not mistaken in inviting for the time being to store raw data, pending major advances. I mean the heuristic of the scope of digital phenotyping on the metaphorical side and a term for a method. The term “phenotyping” nevertheless poses some difficulties insofar as it signals its naturalistic ambition in excluding the singularity dimension of cases and cultural contexts. The singularity of the case from a psychoanalytical point of view is not the singularity of the digital graph that characterizes an individual but the singularity of the meanings the subject can produce to illustrate each trace. Some behavioral parameters can be illuminated by the place they occupy in the economy of the patient’s psychological life. Thus, the analyst may be surprised by the involvement of digital technology in the lives of some patients and may provide additional insight into bio-psycho-social data. There are many approaches to modeling and integrating data. However, the diversity and heterogeneity of these approaches do not work well for large data sets, arguing for the use of these methods on well-constructed and circumscribed data. The ontology-based approach is of particular interest in this regard. Ontology-based modeling is capable of defining knowledge-based entities and allow additional semantics among entities. It is precisely at this stage that the contribution of psychoanalysis could intervene with datascientists. In terms of data use, ontology-based knowledge reasoning can generate new knowledge based on entities and the relationships between entities. Among its benefits is the promise that ontology-based knowledge modeling encapsulates large data sets for digital cataloguing. Recently, ontology has been widely adopted to integrate and analyze large amounts of heterogeneous data in the field of public health and facilitate improved medical diagnosis and treatment (Liang et al., 2019).

Research oriented by psychoanalysis and based on the technical resources of the smartphone constitutes a new and promising field of investigation in the academic domain. The place acquired by psychoanalysis in some universities has indeed allowed the practice of interdisciplinarity (Potier and Putois, 2018; Arcous et al., 2019), research in interaction with neuroscience (Gerber et al., 2006, 2011; Gerber and Peterson, 2006; Bazan, 2007, 2015; Georgieff, 2007, 2010, 2011; Ansermet and Magistretti, 2011; Bazan et al., 2013), followed by evidence of treatment effectiveness from their evaluation (Leichsenring et al., 2004; Leichsenring, 2005; Thurin et al., 2006; Shedler and Westen, 2007; Leichsenring and Rabung, 2008; Shedler, 2010; Fonagy et al., 2015; Thurin, 2017). Psychoanalysis has always had the potential to be irrigated by the life sciences on the one hand and the social sciences on the other. First, through its dialog with the life sciences, which have been combined with information sciences for 50 years and then with contemporary philosophy and social sciences. This specificity makes us particularly sensitive to what can be compartmentalized in certain discourses or devices. This recognition of the division that all knowledge carries also comes from clinical practice. Investing in research allowing for the recognition of this part of impossible should not be considered as hostile to experimental sciences but as a scientific contribution resulting from the psychoanalytical experience itself.

The more development of digital phenotyping techniques is undertaken, the more essential the contribution of psychoanalysis will be on a clinical and practical, scientific, and ethical level. Clinically and practically, because the more patients interact with machines (laptops, prostheses, etc.) the more important it will be to discuss and develop the experience in transference. Regarding online therapies, I shall continue to focus my research on the quality of the subject’s relationship with technical tools, such as the smartphone. The traces of object investments and relationships are data sources unlimited to a strictly behavioral type explanation, where it is necessary to enrich understanding of the data. At this point, ontology-based modeling is central and must be considered. The general issue, beyond the treatment, could be understood from individuation aspect when facing technique (Simondon, 1958), i.e., the conditions of contemporary subjectivation, which psychoanalysis has for a long time linked to man’s relationship with technique (Searles, 1960). Contribution of psychoanalaysis is relevant on the scientific level too, since interdisciplinary research projects can benefit from psychoanalysis in the designing of the study and the plurality of the data sets (evaluation of psychotherapies, epistemological questions, analysis of signifiers, discourse theory, repetition mechanisms, psychopathological revision, dictionary, lexicon, and ontology-based modeling).

Many of the recent meta-analyses I have cited agree that the scope of the real results of unilateral reductionist approaches should be put into perspective. For Ioannidis, I should abandon the research paradigm that considers mental health problems to be mainly brain disorders and direct it toward exploring other roads. This would mean that less emphasis should be placed on identifying the brain’s etiological pathways. If there are no solid and clinically useful biological markers, their perpetual search is futile (Ioannidis, 2019). Finally, I recommend focusing on larger, simple trials with long-term follow-up. These recommendations could lead to a case formulation model (Fishman, 1999; Thurin et al., 2006, 2007; Thurin, 2009, 2012, 2017; Eells and Lombart, 2011; Eells, 2011a,b; Vanheule, 2017).

The case formulation model broadly corresponds to what practitioners refer to as psychopathological diagnosis, but with one notable difference: it is formalized and seeks from the outset to link therapeutic objectives to the inferred causes of the disorders (clinical hypotheses). Case formulation is a dynamic approach to a person’s disorders and problems (Fishman, 1999). Nosographic and psychopathological elements are introduced into an active process involving inferences about the causality of the disorders and problems observed and about the therapeutic process that can bring about change. This model allows a detailed evaluation of the therapy that fits perfectly with psychoanalysis (Thurin, 2017). This formulation could be enriched with the methods allowed by data collection *via* smartphone in a more contextual and targeted use, without claiming exhaustive and large-scale phenotyping, constituting a new source of comparative data. Thus, the crossover of these methods could prove heuristic by focusing on exploiting this rigor in ontological data modeling.

Such formulation requires the identification of a list of problems, a diagnostic basis, the development of an explanatory hypothesis, and the planning of the care pathway, from which it would be possible to test a fine exploitation of the data from a limited and well-informed cohort closely followed by the case formulation method. It would thus be possible to automate certain procedures, to parameterize them in a more targeted manner. Once the case formulation has been developed, it should be tested along with the treatment and revised, especially if the treatment is unsuccessful. Understanding the design of studies including numerical phenotyping according to these recommendations for use would allow a good testing of the evidence in this observation context.

The step of the explanatory hypothesis defined by Eells fits well with what can be measured by collecting data in ordinary life situations *via* mobile phones. Eells suggests that five components of the case should be included: events, the situation that led to vulnerability (biological or other), the person’s assets, the social and cultural context in which they live, and the list of barriers that could hinder treatment (Eells and Lombart, 2011; Eells, 2011a,b). All these elements can be enriched by analysis elements from data collected *via* smartphone. Moreover, there would be an opportunity for practitioners, such as psychoanalysts, to provide *in situ* elements in a much more fluid way, such as facilitating the rating of a therapy (PQS; Jones, 1985) *via* an app dedicated to practitioners. But as mentioned above, the contribution of psychoanalysis would not be limited to this. Researchers oriented by psychoanalysis must also contribute to the enrichment of data and their categorization on the basis of ontologies. Indeed, for enriched data and methods based on machine learning to be beneficial for numerical phenotyping in mental health, rigorous evaluations will be necessary to ensure that the proposed solutions are effective, acceptable, and culturally relevant (Cottler et al., 2015). Also, what psychoanalysis could contribute in this context should be linked to other issues identified in the mental health field, so as to not fall back into a one-dimensional and ultimately reductionist posture.

On the strength of the experiments identified in psychoanalytical research, with verbatim recordings and analyses, there would be, in the rigorous model of case formulation and the targeted integration of the mobile phone into the journey, the opportunity for limited use at the opposite of any reductionist temptation on the part of the patient to their data.

### Advocacy for Networked Case Formulation

Clinical experience, in accordance with psychoanalysis and other models in psychopathology, agree that it is risky to consider mental health problems in a reductionist way. The role of the social sciences in the development of qualitative data is essential. Experience and psychoanalytical theory can provide observations that cross several fields of mental health problems, which can be represented as a network that links several aspects of the reality of mental illness. Each method cannot capture all the elements involved in what is pinned down in mental illness. However, there is also a reticular dimension in psychoanalytic theory that makes it accessible to networked models, which are currently developing in a stimulating way in the field of psychology. The psychoanalytical systems built by Freud, then notably enriched by Lacan, are based precisely on a set of invariant elements ordered around the figure of the network: unbreakable elements (neurons, representations, or signifiers), a structure that takes the form of a network, a fluid that circulates on the network of these unbreakable elements, and a function to regulate this circulation. These elements form a reticular system. Within this reticular system, the network is a real concept for psychoanalysis insofar as it allows us to think of the psyche in a scientific but not reductionist way through a reticular causality, i.e., released from a linear causality (Forest, 2015b). However, the rapprochement between network theory in psychology and the network dimension of psychoanalytical epistemology seeks to highlight the concordance between the approaches with regard to mental health. However, there remain major differences that cannot be developed without straying from the issue of numerical phenotyping. It should thus be noted that the reticular presentation of the psyche is a necessity, as is the presentation of psychopathological problems in these multiple dimensions.

Also, there are currently real criticisms emerging in regard to the reductionist temptation in psychology, whose failures and approximations are pointed out (Borsboom et al., 2019). Holistic research strategies appear as real alternatives for progress in the study of mental disorders. According to Borsboom and colleagues, symptom networks prevent the identification of a common cause of symptomatology with a neurobiological condition; the strength of network relationships depends in part on cultural and historical contexts as well as on external mechanisms in the environment. Taken together, these properties suggest that, if mental disorders are indeed networks of symptoms related to a cause-and-effect relationship, reductionist narratives cannot reach the level of success associated with reductionist disease models in modern medicine (Borsboom et al., 2018, 2019) Thus, for Borsboom and his colleagues, mental disorders do not reflect a single causal factor, but must be conceptualized in terms of complex networks of causal mechanisms (biological, environmental, and psychological).

This holistic methodology is of great interest in fostering the expression of interdisciplinary exchange in mental health research. It also meets one of the dimensions of psychoanalytic theory which is confronted with the need for reticular thinking to account for clinical reality. The network approach marks the difference between “between-subjects” psychopathology networks, whose objective is to explore the general structure of the psychiatric disorder, and “intra-subjects” psychopathology networks, whose objective is to better understand at the individual level how each individual has developed their disorder. From this “within” approach, it is thus possible to create personalized networks for each individual that would allow us to track the evolution of symptoms and better understand why the same factor could lead to the activation of a certain symptom in one person and another symptom in another person (Borsboom et al., 2011).

Also, it seems promising to integrate methods from digital phenotyping to complement and contribute elements to the network approach. This is shown by the literature review conducted by Lydon-Staley and his collaborators (Lydon-Staley et al., 2019). In this paper, the author focuses on the empirical work that merges digital phenotyping data, and in particular experimental sampling data collected using smartphones, with psychopathological theories and network science methodologies. Nevertheless, in the current state of international trade, the global method seems to be highly dependent on the general psychopathology of DSM-5, which poses certain difficulties (Marková and Berrios, 2009; Fellowes, 2017). To take full advantage of these new investigation models and techniques, it is certainly necessary to not only abandon any reductionist temptation on the theoretical level, but also to keep a critical mind in order to avoid imposing normative/normalizing models, *via* data classification and network modeling.

In psychopathology, the network approach aims to study the relationships between the symptoms that contribute to the development and maintenance of one or more psychiatric disorders. It attempts to identify the central symptoms within the network that could trigger the development of other symptoms and that may be the target of management. Indeed, a person’s symptoms cannot be separated from their general functioning, history, and current circumstances in which they are expressed, in short, from their particularities. The environmental aspects, the organization and individual history of the person’s psychological life, are immediately involved. The psychotherapist is involved in their understanding and explanation. The relevance of these elements, which cannot be ignored by a psychotherapeutic approach, is not only a matter of attitude or common sense. It is supported by the large number of clinical, physiological, and epidemiological studies that confirm the chain effects of early history, life events, and current circumstances on a person’s functioning and disorders, as well as the effects of human interactions in their evolution. I can no longer speak of a general cause of the expression of a disorder, but of a set of causes for which the design of actions is adjusted and prioritized according to the characteristics of each case and its potential modalities of change are essential. This set can be understood from the case formulations (Thurin, 2017). It might seem common sense to consider that clinical issues should be the focus of any research in psychopathology. Thus, this multiplicity of causes needs to r work on a set of data that is well categorized and enriched. To reach its full potential, digital phenotyping for mental health should be developed in many directions. One of these directions is data modeling. The data important for digital phenotyping are rich, which poses great challenges for data presentation.

Academic psychoanalysis can find in the case formulation model and network theory the opportunity to participate in interdisciplinary research in the clinical field. The theory of networks can be endlessly enriched through interdisciplinary exchanges and in particular by the contribution of psychoanalytical data. Engaging in this promising research is all the more important since psychoanalysis has much to contribute to the multiple effects of the digitalization of the world.

### Limitations and Perspectives

According to the literature on digital phenotyping, integrating these data into clinical practice in an ethically sound, legally acceptable form for patient privacy will be a major challenge for the success of these approaches (Martinez-Martin et al., 2018). Since existing ethical and regulatory frameworks for mental health care delivery do not clearly apply to digital phenotyping, it is essential to examine its potential ethical, legal, and social implications. It seems that the work on methods based on this type of data and the way they are collected must be done in interdisciplinary interaction (Montag et al., 2019). The quality of the data depends on it (Vaidyam et al., 2019). Practical measures to make this type of research program realistic include the development of technology platforms focused on scalability and equity, the establishment of shared data repositories and common data standards, and the promotion of multidisciplinary collaborations among clinical stakeholders (including patients), computer scientists, and researchers (Huckvale et al., 2019).

Moving toward using the potential of smartphones for research in psychopathology can also aim at a less global ambition. Building ontologies from the close observation of a well-constituted cohort, followed and formulated as cases, could be one of the unexplored avenues of digital phenotyping methods. Interdisciplinary dialog should be initiated to identify limitations already observed and problems that may have been identified in other areas. Finally, it must be possible to launch heuristic and targeted programs in contexts that do not engage large-scale prevention from the outset. There are many arguments in favor of this caution, as indicated by a study to analyze a small number of stand-alone apps currently available on mobile platforms that have been evaluated (Byambasuren et al., 2018). The poor overall quality of evidence on efficacy greatly limits the prescriptibility of health applications. Health applications must be evaluated: systematic reviews should include sensitivity analysis of trials with a high risk of bias to better summarize the evidence and should follow relevant reporting guidelines, as the author says.

In a recent issue of *The Lancet*, an editorial was published that questions the reality of digital medicine in its evolutions and promises (Lancet, 2018). It is noted that research, particularly in the field of AI, has remained focused on the results expected by Machine Learning methods, while actual results and clinical applications are not as conclusive and do not go much further than the rhetoric of the promise. Studies on the effectiveness of the use of these techniques would still have to be produced. However, there is a need for standards, not only for data security and use, but also for the clinical effectiveness and cost-effectiveness of digital medicine according to *The Lancet*. The American Psychiatric Association does suggest a model for evaluating digital applications, taking into account safety issues and evidence of effectiveness, but notes that most applications do not have clinical evidence to support their claims. Without a clear framework to differentiate effective digital products from commercial opportunism, companies, clinicians, and policy makers will have difficulty providing the level of evidence required to accomplish the potential of digital medicine. The risks of digital medicine, in particular the use of AI in health interventions, are therefore a concern. Failure to continue to evaluate health interventions involving digital technology presents great risk to patients and health systems according to *The Lancet’s* analysis.

According to countries, environment makes these innovations more or less susceptible of maximizing these opportunities: new major strategic and infrastructure models seem necessary for the organization of health care according to studies. This is the conclusion of Sebire and colleagues in their article published in *Digital Medicine* in which it is considered that health systems themselves remain one of the main obstacles to the rapid integration of digital expansion into health (Sebire et al., 2018).

Above all, confidentiality and security issues must be taken into account. On this point, it is worth reading the recommendations and testimonies of the data scientists themselves. For example, O’Neil warns us of the devastating impact of automated decision-making processes, which are most often biased and interfere in areas as crucial for an individual and a democratic society as justice and education, not to mention psychiatry. Her work, based on her experience as a data scientist, constitutes a precise analysis with alarming conclusions (O’Neil, 2016). She wishes to alert data scientists themselves and encourage them to take responsibility for the codes they produce and to acknowledge that they have an impact and what it means. Despite their apparent opacity, it is essential to be explained what each mathematical formula is intended to achieve in an algorithmic procedure and therefore in psychopathology projects that could be based on such models. It must be possible to challenge them if the issue of consent is a concrete one. It is therefore essential not to blindly accept the results because they are based on mathematical truth. It is a question of guaranteeing the possibility of auditing the algorithms that govern. The right data science, according to O’Neil, does not speak for itself: we must be careful about the effects it produces and make sure they do not create suffering for a category of population.

Questions about data ownership (for example, who owns your digital phenotype?) and how it will be used (for example, for health care information or for commercial purposes) must be explored without compromise and with complete vigilance.

From these difficulties, it is possible to reflect on recommendations and guide research questions potentially accessible to phenotyping techniques, such as artificial intelligence applications.

As a part of the Light4deaf project, we were able to conduct pair interviews on a large cohort of patients with rare diseases involving deafness and blindness (Potier and Putois, 2018; Arcous et al., 2019). Retrospective analysis of the data will make it possible to identify the subjective problems that the disease induces, both socially and psychologically. This dual orientation will offer the possibility of an extension of the results of this research in the modeling of an app dedicated to patient follow-up. The time required to model this project on a specific population and monitor it for several years is in line with the methodological and ethical prudence considerations with which these new methods are confronted.

## Conclusion

The field of mental health is particularly complex. Subject to controversy, these show the audacity, interest, and limitations of many studies. On the epistemological level, a look at the stakes of the new directions taken by digital phenotyping projects calls for a methodology clarification, in particular the use of psychopathological references as a potential bias in research. On this point, it seems that the debates around the DSM-5 are at the heart of the challenges of these research systems.

The orientation axis proposed by the RDoC should be modified or rather opened up to subjective dimensions and data from the social sciences. This article argues for this heuristic complexity and joins the many reservations about any reductionist temptation in this field. Like neurobiological models, trait models make intuitive sense to researchers. Like neurobiological models, trait models capture an element of psychological dysfunction, but they cannot explain psychological disorders in all their complexity. The network model not only provides an important context for understanding the strengths and limitations of neurobiological models but also trait models like HiTOP. Also, the contribution of psychoanalysis to the issues related to these new research opportunities testifies first of all of a relationship measured to the technique and informed by the clinic and interdisciplinary dialog. Many contributions shed light on the contemporary specificities of discomfort in civilization or propose to explore new possibilities for analysis using technical devices for more than 50 years. As a result, psychoanalysis offers clinical expertise that can be translated into research issues involving smartphone data. On this point the research must be without compromise and in the rigor of what a case formulation indicates, starting from the clinical basis, the single case, or in small groups, in order to avoid ideological bias, marketing, and potential reductionism. The scope of engagement in this research is therefore also ethical. There is no doubt that these techniques will revolutionize disease investigation and patient follow-up; hence it is essential to support these developments and participate in them with a temperament, which “requires two basic qualities: optimism in attempt, criticism in work” (Freud, 1884).

In the context of these new ambitious projects, network theory has emerged as a fruitful theoretical proposal that could be used by psychoanalysis to explain the results of its experience and participate in complex projects arising from the clinical field. The current participation of teams of researchers oriented by psychoanalysis therefore appears promising. Retrospective analysis of data from Light4deaf research interviews may allow a heuristic modeling of a proposal to integrate smartphones into the monitoring of genetic developmental diseases and mental health problems specifically identified since this study. This is one of the results that can be expected from a thorough qualitative analysis prior to the implementation of a project involving embedded technologies, such as smartphones.

Many studies will emerge in this context, this article wishes to lay some theoretical foundations and some heuristic hypotheses testifying of the interest of the participation of psychoanalysis in the development and support of these new orientations in mental health.

## Author Contributions

The author confirms being the sole contributor of this work and has approved it for publication.

### Conflict of Interest

The author declares that the research was conducted in the absence of any commercial or financial relationships that could be construed as a potential conflict of interest.
